# Availability and utilization of medical devices in Jimma zone hospitals, Southwest Ethiopia: a case study

**DOI:** 10.1186/s12913-016-1523-2

**Published:** 2016-07-19

**Authors:** Beyene Wondafrash Ademe, Bosena Tebeje, Ashagre Molla

**Affiliations:** College of Health Sciences(CHS), Population and family Health Departement, Jimma University, Jimma, Ethiopia; College of Health Sciences(CHS), Nursing Departement, Jimma University, Jimma, Ethiopia; College of Medicine and health sciences, School of nursing, Bahirdar University, Bahirdar, Ethiopia

**Keywords:** Medical devices, Availability, Utilization, Functionality, Southwest Ethiopia

## Abstract

**Background:**

Health systems throughout the world, whether in developed or developing countries, are struggling with the challenge of how to manage health-care delivery in conditions of resource constraint. The availability and utilization of various health care equipments at all levels of the health care system has been emphasized for effective and efficient service delivery. In Ethiopia lack of proper management of medical equipment limited the capacity of health institutions to deliver adequate health care. The main objective of this study was to assess availability and utilization of medical devices and identify reported reasons that affect availability and utilization of medical devices among hospitals in Jimma Zone.

**Methods:**

A cross-sectional multiple case-study using mixed quantitative and qualitative methods was used. Three hospitals of Jimma Zone were included in the study. Adapted and pre-tested structured English version checklist for availability and utilization of medical equipment and document review as well as interview guide for in-depth interview were used for data collection. Data were collected by observation of availability of the devices, interviewing selected professionals and document review of health care services using devices in the study hospitals. Data were analyzed using SPSS 16.0 statistical software. Descriptive analysis was made to determine the availability and functional status of medical devices. For qualitative part responses were transcribed, categorized and thematically analyzed.

**Results:**

Observation and interview using checklist showed that 299 medical devices were available in the three hospitals among which, 196 (65.6 %) of them were available in Jimma University Specialized Hospital whereas, 57 (19.0 %) and 46 (15.4 %) were available in Limu Genet hospital and Shenen Gibe hospital respectively. Among 196 available medical devices in JUSH, 127 (64.8 %) were functional and the rest; 63 (32.1 %) and 6 (3.1 %) were not functional and not in use respectively. Similarly, 28 (60.9 %) and 30 (52.6 %) of the devices in Shenen Gibe hospital and LGH respectively were functional.

**Conclusion:**

More than a third of medical devices in the three study hospitals were not functional. Purchasing devices with bids and preference for cheap price, lack of training on how to operate devices, less sense of accountability, power interruption, staff work overload and lack of maintenance experts, and inappropriate referral system were among the reported reasons for influencing availability and utilization of medical devices.

**Electronic supplementary material:**

The online version of this article (doi:10.1186/s12913-016-1523-2) contains supplementary material, which is available to authorized users.

## Background

Eventhough medical devices are indispensible for all aspects of healthcare many appropriate technologies are inaccessible to the majority of people who need them, particularly in low and middle-income countries [1]. Many people in developing countries do not have access to health technologies, even basic ones. These technologies include life-saving medicines, such as antiretroviral for HIV/AIDS, as well as life-enhancing medicines, such as antiasthma medications that help stop asthma attacks and improve breathing.

Essential medical products must be available at all times at the appropriate level within the health-care system. [2]. Improvements in the health of much population are associated with the improved ability to predict, prevent, diagnose and cure many illnesses, and to alleviate functioning problems using treatments and technologies [3]. A well-functioning health system ensures equitable access to essential medical products, vaccines and technologies of assured quality, safety, efficacy and cost-effectiveness, and their scientifically sound and cost-effective use [4].

Health systems throughout the world whether in developed or developing countries, are struggling with the challenge of how to manage health-care delivery in conditions of resource constraint [5]. Lack of working equipment has a devastating effect on healthcare in resource-poor settings. It is often said that most of the medical equipment in the developing world is broken with estimates ranging up to 96 % out of service. More than 50 % of the laboratory and medical equipments in resource-poor settings are not in service [6].

In Ethiopia, lack of proper management of medical equipment has limited the capacity of health institutions to deliver adequate health care. It is estimated that only about 61 % of medical equipments found in Ethiopian public hospitals and other health facilities are functional at any one time. Medical equipment management defines organization and coordination of activities that ensure the successful management of equipment related to patient care in a health facility [7, 8].

However, there is limited evidence in availability and utilization of medical devises and technologies in Ethiopian hospitals in general, and in hospitals of Jimma zone in particular. Therefore, this study was designed to identify the reported reasons that contributed to availability as well as utilization of medical devices in the respective hospitals. This will help hospital administrators and other stakeholders to create awareness as a means of resolving problems through communication and to take informed actions for better health care delivery.

## Methods

The study was conducted from August 6–30, 2013 in all three hospitals found in Jimma Zone, Oromia regional state, Southwest part of Ethiopia. Based on the 2007 census, the Zone has a total population of 2,486,155. The three hospitals in Jimma Zone are Jimma University Specialized Hospital (JUSH), Shenen Gibe Hospital (SGH) and Limu-Genet Hospital (LGH) [9].

JUSH and SGH are found in Jimma town which is located 350 km southwest of Addis Ababa. JUSH is the only teaching and referral hospital in southwest part of the country. JUSH has bed capacity of 450 and a total of more than 750 staffs of both supportive and professional. It provides services for approximately 9000 inpatient and 80,000 outpatient attendances a year, coming from the catchment population of about 15 million people. SGH is newly established hospital located on other end of the city and serves communities of the city and surrounding areas. LGH is a district hospital found in Limu-Genet town that is located 80 km south of Jimma town.

Cross-sectional case study with mixed methods including in-depth interview, observation and document review was conducted. Observation of devices at 16 units, document review; and nine in-depth interviews were conducted. Adapted structured English version checklist for availability and utilization of medical equipments from the report by Indian Employee’s state insurance corporation [10] document review, and interview guide for in-depth interview were used for data collection. The checklist was adapted to be suited for district and referral hospitals. This was done by considering the services available at referral and district hospital in Ethiopia. Some of the medical devices from the list were omitted to study district hospitals since those medical devices were not available due to the unavailability of a particular service in the hospital e.g. major devices used for ICU were not included since the ICU service is not available in district hospitals.

The checklist include medical device lists which should be present in typical district or referral hospital and number of available devices with level of functionality and frequency of monitoring. The availability of the medical devices was checked by observation of different units using the checklist. The units where observation conducted were radiology, ophthalmology, laboratory, Operation Theater and Central sterilization, maternity and newborn care, and Intensive care unit (ICU). ICU observation was conducted only on the specialized teaching hospital since the service is available in JUSH among the study hospitals. Patients’ registration books and procedure registration books were reviewed. Functional status of the equipments were determined as “functional” if it was on use during data collection, “not functional” if it stopped functioning and “not in use” if it was not ever used since the device were procured or donated.

Data were collected from different departments of the study hospitals by using observation checklist, interview guide & document review. Key informants for in-depth interview were selected professionals (matrons, hospital Chief Executive Officers (CEO), and medical device store managers).

All data collectors had BSc. qualification in health related and/or bio-medical engineering field. The data collectors were those who have no role in medical device control and management responsibility to avoid data collectors’ bias. Researchers supervised data collection process and conducted in depth interview. Two days of training for data collectors on the objectives of the study and how to approach and handle questions was given by investigators.

Different data quality control measures were taken. Study tools (Additional file 1) were adapted from similar studies and modified based on the objective of the study and context of the study area to assure the content quality of the data. The experts assured the content validity of the tool. Training of the data collectors and close supervision during data collection were conducted. The checklist was pre-tested at Woliso St. Luke Hospital. Operation theatre, Inpatient, Device store, radiology department, Central sterilization service and other appropriate unites of Woliso St. Luke Hospital were observed using the prepared checklist. Additionally the coordinators of each observed unit, CEO, matron and medical director of the hospital were interviewed using the prepared in-depth interview guide. Necessary modifications (adding medical device on the list for a unit or taking to another unit) were made on data collection tools after the pretest. The quality of the data was also assured by explaining unclear ideas during data collection, checking completeness and consistency of filled checklist and performing exploratory analysis.

Qualitative data were collected through in-depth interview using in-depth interview guide. The interview guide focus on source of medical equipments, utilization and functioning of medical devices, human resource to manage medical devices, procurement, quality assurance mechanisms, and measures in prevention and control of occupational hazard. A total of 9 interviews were conducted in Amharic language. During interview the response was tape-recorded and field note was taken.

The quantitative data were analyzed using statistical package for social sciences (SPSS) version 16.0. Descriptive analysis was done to describe frequency and percentage of the available medical devices and their functional status in each institution. The qualitative data were analyzed after the responses were transcribed for every interviewee separately. Then the transcribed data translated to English and categorized and analyzed thematically. Ethical clearance was obtained from ethical review board of college of public health and medical science of Jimma University. After getting ethical clearance, written permission was obtained from Jimma zone health office administration. Verbal informed consent after explaining confidentiality of data was obtained from CEO of each study hospitals and each study participant.

## Results

### Availability of medical devices

Two hundred ninety-nine medical devices were available in the three hospitals among which, 196 (65.6 %) of them were available in JUSH whereas 57 (18.1 %) and 46 (15.4 %) were available in LGH and SGH respectively (Table 1).

**Table 1:** Frequency distribution of available medical devices in the hospitals found in Jimma Zone, South-west Ethiopia: August, 2013

Medical device at different departments	Number of available medical devices			
		JUSH	SGH	LGH
Radiology	X-ray	2	1	2
	Ultrasound	2	1	2
Dental	Dental X-ray	3	0	0
	Chair	10	0	2
	Sterilizer	4	0	2
	Amalgamate	1	NA	NA
Ophthalmology	Ophthalmoscope	3	0	2
	Refractometer	1	0	0
	Refraction set	2	NA	NA
	Tonometer	4	NA	NA
	Anesthesia machine	2	NA	NA
	OR table	4	NA	NA
Laboratory	Microscope	19	5	4
	Centrifuge	4	2	3
	Hotplate	0	1	0
	Cell counter	3	1	1
	Refrigerator	8	1	5
	Deep freezer	2	1	0
	Bio-safety cabinet	5	0	0
	Water bath	3	0	0
	Laminar flow	2	0	0
	Incubator	3	0	1
	Weighting balance	1	0	0
	ELISA Reader	2	0	0
	Immuno Assay System	4	0	0
	Chemistry analyzer	7	2	1
	CD4 counter	2	NA	NA
	PCR	1	NA	NA
Operation theater	Electrosurgical unit	3	2	0
	Autoclave	3	4	7
	Ultrasound machine	0	0	2
	ECG machine	0	1	1
	Patient monitor	3	2	3
	Anesthesia machine	3	3	2
	OT Table	3	2	2
	Oxygen concentrator	4	1	1
	OT light	3	3	2
	Suction machine	6	4	5
	Fluoroscope	1	NA	NA

NB. Many micropipettes are available in JUSH where as 3 and 5 are available in SGH and LGH respectively

*NA* Not Applicable

**Fig. 1:**
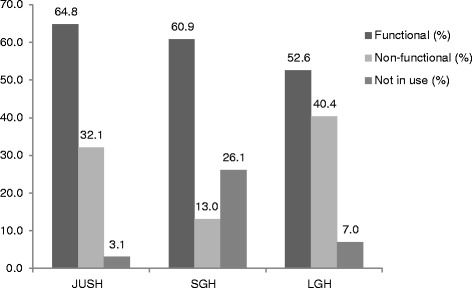
Showing function status of devices in the three hospitals

Among 196 available medical devices, 127 (64.8 %) were functional and the rest; 63(32.1 %) and 6 (3.1 %) were not functional and not in use respectively. Twenty eight (60.9 %) and 30 (52.6 %) of the devices in Shenen Gibe hospital and LGH respectively were functional (Table 2 and Fig. 1).

**Table 2:** Frequency distribution of functional status of medical devices in the hospitals found in Jimma Zone, South-west Ethiopia, August, 2013

Medical device	Functional status								
	JUSH			SGH			LGH		
	Functional	Not functional	Not in use	Functional	Not functional	Not in use	Functional	Not functional	Not in use
X-ray	2	0	0	1	0	0	2	0	0
Ultrasound	2	0	0	1	0	1	1	1	1
Dental X-ray	2	0	1	NA	NA	NA	NA	NA	NA
Chair	5	5	0	NA	NA	NA	1	1	0
Sterilizer	2	2	0	NA	NA	NA	2	0	0
Amalgamate	0	1	0	NA	NA	NA	NA	NA	NA
Ophthalmoscope	1	2	0	NA	NA	NA	1	1	0
Refractometer	1	0	0	NA	NA	NA	NA	NA	NA
Refraction set	1	1	0	NA	NA	NA	NA	NA	NA
Tonometer	1	3	0	NA	NA	NA	NA	NA	NA
Anesthesia machine	3	2	0	1	1	1	1	0	1
OR table	2	2	0	NA	NA	NA	NA	NA	NA
Microscope	10	9	0	2	1	2	3	1	0
Centrifuge	4	0	0	1	0	1	3	0	0
Hotplate	0	0	0	1	0	0	0	0	0
Cell counter	2	1	0	1	0	0	0	1	0
Refrigerator	7	1	0	1	0	0	4	1	0
Deep freezer	1	1	0	1	0	0	NA	NA	NA
Bio-safety cabinet	4	1	0	NA	NA	NA	NA	NA	NA
Water bath	3	0	0	NA	NA	NA	NA	NA	NA
Laminar flow	2	0	0	NA	NA	NA	NA	NA	NA
Incubator	3	4	0	NA	NA	NA	0	1	0
Micropipette	NA	NA	NA	3	0	0	3	2	0
Weighing balance	1	0	0	NA	NA	NA	NA	NA	NA
ELISA reader	2	0	0	NA	NA	NA	NA	NA	NA
Immuno Assay System	4	0	0	NA	NA	NA	NA	NA	NA
Chemistry Analyser	5	2	0	1	0	1	1	0	0
CD4 counter	0	2	0	NA	NA	NA	NA	NA	NA
PCR	1	0	0	NA	NA	NA	NA	NA	NA
Electrosurgical unit	2	1	0	2	0	0	NA	NA	NA
Autoclave	2	1	0	2	1	1	1	6	0
Fluoroscope	1	0	0	NA	NA	NA	NA	NA	NA
ECG machine	0	1	0	1	0	0	0	1	0
Patient monitor	6	4	0	0	0	2	0	2	1
OT Table	3	0	0	2	0	0	2	0	0
OT light	3	0	0	3	0	0	1	1	0
Suction machine	3	5	0	2	3	1	2	4	0
Infant warmer	2	0	1	1	0	0	0	0	1
Fetal Doppler	1	0	0	NA	NA	NA	NA	NA	NA
Pulse oxymeter	17	4	4	0	0	0	0	0	0
Vacuum	3	0	0	0	0	2	1	0	0
Incubator	0	4	0	NA	NA	NA	NA	NA	NA
Humidifier	7	0	0	NA	NA	NA	NA	NA	NA
Defibrillator	4	0	0	NA	NA	NA	NA	NA	NA
Mechanical ventilator	3	4	0	NA	NA	NA	NA	NA	NA
Oxygen concentrator	0	4	0	1	0	0	1	0	0
Total	127	63	6	28	6	12	30	23	4

The participants in qualitative study were aged from 24 years to 35 years. The qualitative part of this study identified the main sources on the medical devices; the main reported factors for the good or poor utilization, and monitoring of these devices were addressed.

Thematic areas of the in-depth interview result include major sources of medical equipments of the hospital, appropriate medical devices utilization and functionality, presence of sufficient human power to utilize/functionalize the existing medical devices, the involvement of appropriate professionals in the order, purchase and procure and utilize the medical devices, quality assurance methods of medical devices, solutions taken for non-functioning equipments, measures taken by the hospital to prevent and control occupational hazard, presence of interruption of health service due to failure of medical device, reason for the interruption and of medical device, measures taken during the interruption of the service, presence of responsible person for the equipment, presence of policy/guideline document for the equipment utilization, how maintenance was made in case the device fails to function, and frequency of equipment monitoring (Table 3).

**Table 3:** Thematic areas for the in-depth interview conducted for the availability and utilization of medical devices, Jimma zone southwest Ethiopia, August 2013

1. Major sources of medical equipments of the hospital
2. Appropriate medical devices utilization and functionality
3. Presence of sufficient human power to utilize/functionalize the existing medical devices
4. The involvement of appropriate professionals in the order, purchase, is procure and utilize the medical devices
5. Quality assurance methods of medical devices
6. Solutions taken for non-functioning equipments
7. Measures taken by the hospital to prevent and control occupational hazard
8. Presence of interruption of health service due to failure of medical device
9. Reason for the interruption and of medical device
10. Measures taken during the interruption of the service
11. Presence of responsible person for the equipment
12. Presence of policy/guideline document for the equipment utilization
13. How maintenance was made in case the device fails to function
14. Frequency of equipment monitoring

Key informants reflected that major sources of equipments or medical devices were from both governmental and donation from non-governmental organizations including FMHACA, regional Health bureau, zonal and town Health office, ICAP Ethiopia, programs/projects (e.g. CD4 machine by HIV related programs) and Human Bridge; some chemicals and reagents were purchased from private companies by the hospital budget.

The existing procurement process had contribution for getting quality medical device. Key informants on in-depth interview in JUSH outlined that there is a problem not only the way of utilization but also the type of equipment purchased such as available stethoscope and blood pressure apparatus. Stethoscope purchased is not a type of what the staff request and needed. They also reported that JUSH, the equipments is purchased with bids that requested cheap price which in-turn affect the quality. This problem is mainly related with the system of purchasing/ procurement and the way bids were done. As one key informant stated “*If we need quality equipment, we have to pay more; the equipment bought with low price will be out of use immediately; for example, if we see digital blood pressure apparatus, after reading once, it will start to have false reading*”.

The study identified the presence of different approaches of medical device procurement process. For instance one of the female respondents said “*purchasing is done based on the request and specification of each department. Procurement committee is composed of different professionals including matron, auditors, CEO, and pharmacist. Different devices are procured from clear bid announced by the university (JU) and the one with cheap price will win. Whatever the quality of the device the cheap one is preferred. Sometimes poor quality may come.*”

One female interviewee from one of the district hospitals stated “*there was CBC machine, but it takes more than a year to install by the company which should come from Addis Ababa by that time the reagent expired. Chemistry machine is available but the company did not come to install*.”

### Functional status of medical devices

In JUSH among 196 available medical devices, 65.13 % were functional and the rest; 32.31 % and 3.08 % were not functional and not in use respectively. Only 62.2 % and 51.7 % of the devices in SGH and LGH respectively were functional.

Among the causes for non-functionality of the equipments, the type of equipment purchased such as available stethoscope and blood pressure apparatus were not to the standard which is needed since the equipments were purchased with bids that requested cheap price which in-turn affect the quality. This problem was mainly related with the system of purchasing/ procurement and the way bids are done. One female interviewee reported “*purchased medical equipments start functioning and they fail soon. Being in poor country should not be a reason for using medical device which is below the standard/of poor quality*”.

The attitude of staffs in taking care of the equipment and being dependent on donation were other factors affecting utilization and functionality of devices. The sustainability of medical device supply is also another factor in implementing day to day activities such as the implementation of nursing care standard in this hospital. When staffs are recruited and increase the human power, the equipment and supply should be increased.

The plan of the hospital depends on the number of patient care/service not on the number of students who come for training in the hospital. Many individuals may use the machine which leads to over or inappropriate utilization of the device/supply. While some medical devices are installed, training may be given for one person but this person may leave for different reasons, so another person without training may take over the responsibility to operate the machine which also affects the function of the device. “*X-ray machine is usually become non-functional, the reason is not clearly known but I think the way it is used because the machine is modern and professionals not used to it during their training*” of the male key informants from district hospital explained.

### Monitoring of medical devices

Thirty one medical devices that were supposed to be in hospitals particularly in specialized referral hospitals were not available in all the three study hospitals (Table 4). Of all available devices, only 3 (1.01 %) of devices were monitored either once or twice per year in the three hospitals. Ethiopian Health and Nutrition Research institute (EHNRI), Oromia regional heath office (OHB) and Ethiopian Ray Authority (ERA) are the agencies who monitored those devices (Table 5). Participants of the in-depth interview at all hospitals expressed that there is no monitoring and quality assurance methods. In relation to maintenance issue at JUSH, there is biomedical unit but it is not functional. Some devices were maintained by the company and having warranty where as in Shenen Gibe district hospital monitoring is non-existent, only inventory is done twice per year.

**Table 4:** Medical devices which are not available in all the three hospitals of Jimma Zone, southwest Ethiopia: August, 2013

Departments in the hospital	Name of the medical device
Radiology	Portable X-ray
	CT Scan
	MRI
Inpatient	Traction unit
	Hemodialysis machine
Laboratory	Cell separator
	Urine analyzer
	Plasma thawing Bath
	Platelet aggregator
	Tube sealer
	Microtome
	GenXpert
Operation theater	Heart and Lung machine
	Arterial Blood Gas Analyzer
	Ultrasonic Washer
	IABP (Intra-aortic Balloon Pump)
	TMT machine
	PFT machine
	Cardiac monitor
	Ventilator
	Insufflators
	Endoscope
	Syringe and infusion pump
Maternity and neonatology	Phototherapy
	Infant resuscitator
ICU	ACT machine
	Blood and fluid warmer
	Electromyogram machine
	Pacemaker
	Cardiac monitor
Total	31

**Table 5:** Frequency distribution of monitoring of available medical devices in the three hospitals found in Jimma Zone, South-west Ethiopia. August, 2013

Medical device	Annual frequency of monitoring and agency who monitor					
	JUSH		SGH		LGH	
	Frequency	Agency	Frequency	Agency	Frequency	Agency
X-ray	None	–	Twice	OHB	Once	ERA
Cell counter	None	–	None	–	Twice	EHNRI
Bio-safety cabinet	Once	ENHRI	None	–	None	–

Quality assurance was practiced only in laboratory departments especially for kits. LGH hospital has no its own maintenance professionals; technician from other institutions tries to fix the problem, if not possible use alternative new machine, if impossible patients will be forced to go to private organizations provided that they can afford payment.

Other reported factors affecting availability of medical device in this hospital includes: unavailability in the market, and inadequate budget. About utilization of devices dissatisfaction of staff, negligence, less sense of accountability, staff overload and being a teaching hospital are among the factors affecting proper utilization or functionality of devices were reported factors. Being the referral hospital, some medical devices may also be overused. Inappropriate referral system is also contributing to shorten the life of devices, meaning the hospital is intended to see only referral cases not to provide primary healthcare activities, which increase load on equipments and machines.

The two district hospitals also shared similar problems for instance in SGH, there were some devices such as ultrasound machine, which was not in use because there was no professional to operate it. Miss-utilization of equipments sometimes existed, for example, blood pressure cuff lost, broken and become non-functional which seems due to negligence and poor quality of the device. Lack of motivation for those staff members who were taking care of devices may also be contributing factor. The hospital also has no maintenance experts.

In relation to motivation strategies, different views were seen. Recognition, educational opportunity and hazard allowance was viewed as important motivation measures which were practiced little. One female interviewee said “*most staffs complain that the work they are doing and the payment is mismatching. Sometimes they may make devices disabled due to their dissatisfaction.*” Another male interviewee reported that “*medical equipments like X-ray have become non-functional frequently but recently, the hospital obtained digital X-ray which is working properly and never been non-functional*.” Over utilization of devices is also a problem which shortens the lifespan of devices. There were times at which devices failed to function and not be maintained.

Guidelines and policy for the equipment use is non-existent. One female interviewee from one of the district hospital said “*There are no guideline/ policy for the hospital which describes on how to use the medical device. The hospital is usually evaluated by the EHRIC standard but the result is very low. No measure was taken as a result because there is no Bio-medical engineer for this hospital. It was asked to hire but there was no permission because, the structure of the district hospitals do not allow having bio-medical engineers*”. In relation to occupational hazard such as excessive ray exposure, there is monitoring of the amount of ray the professionals are exposed to but no measure was taken. No risk prevention other than training given on infection prevention was reported in this study.

Documents were reviewed to identify services provided or interrupted due to device functionality. Reviewing documents at different departments of JUSH revealed that on average 350 deliveries per month at maternity, 25 different surgeries per day at OR, 65 patients per day at Ophthalmology departments, 300 patients per day at lab, 42 patients per month at ICU department, 150 patients/day at X-ray, 30 patients/day at dental and 70 neonates/month at neonatal unit obtained service. The main reason for service interruption was mainly due to power interruption and failure of devices to function. Operation room at one of the district hospital was not functioning mainly due to generator/power failure.

## Discussion

Eventhough the Ethiopian health institutions standards for hospitals indicated equipments to be available in specialized hospitals, valuable medical equipments were missed. This unavailability may be explained by the fact hospitals do have limitation on purchasing such expensive medical devices and/or inability of donors to donate such expensive equipments. This will limit the healthcare service of patients especially whose medical diagnosis needs these medical devices for diagnosis or intervention [7, 11, 12].

The major sources of equipment in the study hospitals were through purchasing and donation. This study is supported by study conducted by Frank Eric Zomboko et.al that showed many of medical equipments in developing countries are donated [13].

The functionality of medical devices (34.9 % not functional and not in use) in this study finding is comparable with the study conducted by Franc Eric Zomboko et.al which showed that 40 % of the medical devices in the developing world were out of services [12, 13].

Among the causes for non-functionality of the equipments, the type of equipment purchased is the one with lowest price since the equipments were purchased with bids that requested cheap price which in-turn affect the quality. This problem is mainly related with the system of purchasing/procurement and the way bids were done. Attitude of staffs in taking care of the equipment, sustainability of medical device and supply, lack of training on handling medical devices and being dependent on donation were other factors affecting utilization and lasting functionality of devices is also another factor in implementing day to day activities such as the implementation of nursing care standard in this hospital. When staffs recruited and increase the human power, equipments and supply should be increased. Some of these factors are also described by the report on medicines and medical supplies and in the study done by P. Th. Houngbo et.al [14, 15]. The variation in these studies might be due to the difference in the setting and scope of studies.

The study conducted in Benin on factors that affect medical device access and utilization in selected hospitals and health centers identified; unavailability of medical devices, lack of spare parts of medical devices, equipment service manuals and lack of equipments for implementation activities [16].

Being not available in the market, inadequate budget, lack of spare parts, and dissatisfaction of staff, negligence, less sense of accountability, staff overload, and inappropriate referral system were factors that affect availability and utilization of medical devices identified as factors in other previous studies [14, 15, 17].

Monitoring of medical devices in the hospitals was very poor in which only 1 % of the devices are monitored even though the frequency of monitoring is less. This may be due to absence of hospital policy/guideline which promotes monitoring of devices regularly. Since it is difficult to procure cost-effective equipment for health care sectors by low income countries [16] sufficient advisory and supervisory capacity needs to be developed at all health facilities for maintenance and monitoring which operated by skilled professionals.

Even the absence of regular monitoring of the functionality medical devices will have negative impact on patient outcome since medical devices can create risk to patients and staff [8] which obligate the hospital administrators to give attention on regular monitoring and maintenance plan.

### Limitation of the study

Generalization to other hospitals is not possible due to limited sample size. Since the study includes only three hospitals (one referral and two district hospitals), it will be difficult to generalize to all referral and district hospitals.

## Conclusion

Significant numbers of medical devices which should be available in the referral hospitals were not available. Additionally, more than a third of the medical devices in the three study hospitals were not functional and not in use. The major sources of medical devices were through procurement, donation and through academic programs and projects. Level of involvement of different professionals in procurement of medical devices varied from hospital to hospital.

The way of purchasing of medical devices in which devices were being purchased with bids that requested cheap price; attitude of staffs in taking care of the equipment; limited capacity to purchase quality devices with reasonable price; being dependent on donation; lack/absence of spare-parts for donated medical devices; mismatch between demand and supply (number of staff and supply); inappropriate utilization and/or over-utilization of devices, misuse or intentionally disabling medical equipment; lack of training while medical devices installed; taking responsibility to operate machines without appropriate training; being not available in the market; power interruption, lack of maintenance staff; insufficient or no motivation for professionals; absence of standard to monitor devices; absence of structure which allow to recruit bio-medical engineers for district hospitals; less sense of accountability and staff work overload were among the reasons mentioned by the participants that affect availability of and proper utilization of devices.

Inappropriate referral system was also reported for the contribution to shorten the life of devices by increasing the workload on some devices. There was very low monitoring of medical devices in all the three hospitals. There was also no sufficient maintenance practice of medical devices and district hospitals had no their own maintenance expert.

## Abbreviations

CBC, complete blood count; CEO, Chief Executive Officer; CT, computed tomography; EHNRI, Ethiopian Health and Nutrition Research Institute; ESA, Ethiopian standard agency; FHS, future health system; FMHACA, Food, Medicine and Health care Administration and Control Authority; HIV/AIDS, human immunodeficiency virus/acquired immunodeficiency syndrome; HSDP, Health Sector Development Program; ICAP, International Center for AIDS Care and Treatment Program; ICU, intensive care unit; JUSH, Jimma University Specialized Hospital; LGH, Limu Genet Hospital; LMIC, low and middle income countries; MRI, magnetic resonance imaging; OL, operation light; OT, operation table; PPM, planned preventive maintenance; SGH, Shenen Gebe Hospital; WHO, World Health Organization

## Additional file

Additional file 1: Adapted study tools(questionnaire,interview guide and checklist). (DOCX 52 kb)
