# Supplementary material for: Availability and utilization of medical devices in Jimma zone hospitals, Southwest Ethiopia: a case study
Source: BMC Health Serv Res. 2016 Jul 19;16:287. doi: 10.1186/s12913-016-1523-2 (PMC4952070; doi:10.1186/s12913-016-1523-2)
Supplement: Additional file 1: — Adapted study tools(questionnaire,interview guide and checklist). (DOCX 52 kb) [file 12913_2016_1523_MOESM1_ESM.docx]

**Questionnaire**

**Jimma University**

**College of public health and medical sciences**

**Questionnaire to determine the availability and utilization of medical equipments in the hospitals of Jimma Zone, Southwest Ethiopia**

**Verbal Consent form**

**To be read by the interviewer**

Dear respondent, this is a questionnaire is prepared to assess the availability and utilization of medical equipments in the hospitals. So you are kindly requested to respond to the questionnaire honestly so that you are doing great contribution for the better health care provision in Jimma zone and the country at large.

Your cooperation and willingness for the interview is helpful in identifying level of utilization and factors those will affect the utilization of medical devices and related to the subject matter. You have been chosen to participate in the study. Your name and other personal identifications will not be written on the sheet. All information that you gave will be kept strictly confidential.

Are you willing to participate?

Yes

No

Checklist for availability and utilization of medical equipments at **Referral Hospitals** (Radiology, lab, Dental, OR, ICU, Medical, surgical, Gyn/Obs, ophthalmic dept)

| S.No | Equipment name | Availability | | Number | Functioning status And Number | | | Utilization | | Monitoring |  |
| --- | --- | --- | --- | --- | --- | --- | --- | --- | --- | --- | --- |
|  |  | Yes | No |  | Functional | Not Functional | Not in use |  |  | Frequency | Agency |
|  |  |  |  |  |  |  |  | Yes | No |  |  |
|  | X-Ray |  |  |  |  |  |  |  |  |  |  |
|  | CT Scanner |  |  |  |  |  |  |  |  |  |  |
|  | MRI Scanner |  |  |  |  |  |  |  | |  |  |
|  | Mammography |  |  |  |  |  |  |  | |  |  |
|  | Cath Lab System |  |  |  |  |  |  |  | |  |  |
|  | C-Arm Machine |  |  |  |  |  |  |  | |  |  |
|  | Heart & Lung Machine |  |  |  |  |  |  |  | |  |  |
|  | Arterial Blood Gas analyzer |  |  |  |  |  |  |  | |  |  |
|  | Electrosurgical Unit |  |  |  |  |  |  |  | |  |  |
|  | Autoclave |  |  |  |  |  |  |  | |  |  |
|  | Ultrasonic Washer |  |  |  |  |  |  |  | |  |  |
|  | Dental X-Ray Machine |  |  |  |  |  |  |  | |  |  |
|  | Ultrasound Machine |  |  |  |  |  |  |  | |  |  |
|  | IABP (Intra aortic balloon pump) |  |  |  |  |  |  |  | |  |  |
|  | Echocardiography Machine |  |  |  |  |  |  |  | |  |  |
|  | TMT Machine |  |  |  |  |  |  |  | |  |  |
|  | PFT Machine |  |  |  |  |  |  |  | |  |  |
|  | Patient Monitor |  |  |  |  |  |  |  | |  |  |
|  | Cardiac Monitor |  |  |  |  |  |  |  | |  |  |
|  | ECG Machine |  |  |  |  |  |  |  | |  |  |
|  | Defibrillator |  |  |  |  |  |  |  | |  |  |
|  | Anesthesia Machine |  |  |  |  |  |  |  | |  |  |
|  | Ventilator |  |  |  |  |  |  |  | |  |  |
|  | OT Table |  |  |  |  |  |  |  | |  |  |
|  | OT Light |  |  |  |  |  |  |  | |  |  |
|  | Suction Machine |  |  |  |  |  |  |  | |  |  |
|  | Insufflators |  |  |  |  |  |  |  | |  |  |
|  | Endoscope/Laparoscope |  |  |  |  |  |  |  | |  |  |
|  | Syringe & Infusion Pump |  |  |  |  |  |  |  | |  |  |
|  | Infant Warmer |  |  |  |  |  |  |  | |  |  |
|  | Phototherapy Unit |  |  |  |  |  |  |  | |  |  |
|  | Fetal Doppler |  |  |  |  |  |  |  | |  |  |
|  | Patient Bed |  |  |  |  |  |  |  | |  |  |
|  | Pulse Oximeter |  |  |  |  |  |  |  | |  |  |
|  | ACT Machine |  |  |  |  |  |  |  | |  |  |
|  | Tourniquet System |  |  |  |  |  |  |  | |  |  |
|  | Blood and Fluid Warmer |  |  |  |  |  |  |  | |  |  |
|  | Electromyogram Machine |  |  |  |  |  |  |  | |  |  |
|  | Electroencephalogram Machine |  |  |  |  |  |  |  | |  |  |
|  | Bi-Pap Machine |  |  |  |  |  |  |  | |  |  |
|  | Humidifier |  |  |  |  |  |  |  | |  |  |
|  | Holter System |  |  |  |  |  |  |  | |  |  |
|  | Pace Maker |  |  |  |  |  |  |  | |  |  |
|  | Bubble CPAP (Continuous positive airway pressure) System |  |  |  |  |  |  |  | |  |  |
|  | Infant Resuscitator |  |  |  |  |  |  |  | |  |  |
|  | Microwave Diathermy |  |  |  |  |  |  |  | |  |  |
|  | Hot Pack Unit |  |  |  |  |  |  |  | |  |  |
|  | Traction Unit |  |  |  |  |  |  |  | |  |  |
|  | Continuous Passive Motion System |  |  |  |  |  |  |  | |  |  |
|  | Cold Pack unit |  |  |  |  |  |  |  | |  |  |
|  | Ultrasonic Tens System |  |  |  |  |  |  |  | |  |  |
|  | Hemodialysis Machine |  |  |  |  |  |  |  | |  |  |
|  | Continuous renal replacement therapy (CRRT) Machine |  |  |  |  |  |  |  | |  |  |
|  | Donor Couches |  |  |  |  |  |  |  | |  |  |
|  | Microscopes |  |  |  |  |  |  |  | |  |  |
|  | Centrifuge/Cryofuge |  |  |  |  |  |  |  | |  |  |
|  | Hot Plate |  |  |  |  |  |  |  | |  |  |
|  | Cell Counter |  |  |  |  |  |  |  | |  |  |
|  | Cell Separator |  |  |  |  |  |  |  | |  |  |
|  | PH Meter |  |  |  |  |  |  |  | |  |  |
|  | Refrigerator |  |  |  |  |  |  |  | |  |  |
|  | Deep Freezer |  |  |  |  |  |  |  | |  |  |
|  | Bio-safety Cabinet |  |  |  |  |  |  |  | |  |  |
|  | Water Bath |  |  |  |  |  |  |  | |  |  |
|  | Laminar Flow |  |  |  |  |  |  |  | |  |  |
|  | Incubator |  |  |  |  |  |  |  | |  |  |
|  | Urine Analyzer |  |  |  |  |  |  |  | |  |  |
|  | Micropipettes |  |  |  |  |  |  |  | |  |  |
|  | Weighing Balance |  |  |  |  |  |  |  | |  |  |
|  | Plasma Thawing Bath |  |  |  |  |  |  |  | |  |  |
|  | Platelet Agitator |  |  |  |  |  |  |  | |  |  |
|  | Tube Sealer |  |  |  |  |  |  |  | |  |  |
|  | ELISA Reader |  |  |  |  |  |  |  | |  |  |
|  | Immuno Assay System |  |  |  |  |  |  |  | |  |  |
|  | Microtome |  |  |  |  |  |  |  | |  |  |
|  | Refractometer |  |  |  |  |  |  |  | |  |  |
|  | Ophthalmoscope |  |  |  |  |  |  |  | |  |  |
|  | Slit Lamp |  |  |  |  |  |  |  | |  |  |
|  | Keratometer |  |  |  |  |  |  |  | |  |  |
|  | Auto Perimeter |  |  |  |  |  |  |  | |  |  |
|  | Image Capturing system |  |  |  |  |  |  |  | |  |  |
|  | Dental Chair |  |  |  |  |  |  |  | |  |  |
|  | Dental Sterilizer |  |  |  |  |  |  |  | |  |  |
|  | Lithotripsy Machine |  |  |  |  |  |  |  | |  |  |
|  | Lithotripsy Table |  |  |  |  |  |  |  | |  |  |
|  | Uroflowmeter |  |  |  |  |  |  |  | |  |  |
|  | ENT Examination Unit |  |  |  |  |  |  |  | |  |  |
|  | Harmonic Scalpel System |  |  |  |  |  |  |  | |  |  |
|  | Chest Vibrator |  |  |  |  |  |  |  | |  |  |
|  | Fibrillator |  |  |  |  |  |  |  | |  |  |
|  | VDRL Rotator |  |  |  |  |  |  |  | |  |  |
|  | Hormone Analyzer |  |  |  |  |  |  |  | |  |  |
|  | Air Sampler |  |  |  |  |  |  |  | |  |  |
|  | Wax Bath |  |  |  |  |  |  |  | |  |  |
|  | Surgical/Operating Microscope |  |  |  |  |  |  |  | |  |  |
|  | Phaco-emulsification Machine |  |  |  |  |  |  |  | |  |  |
|  | Tissue Flotation Bath |  |  |  |  |  |  |  | |  |  |
|  | Vortex Mixer |  |  |  |  |  |  |  | |  |  |
|  | Transport Incubator |  |  |  |  |  |  |  | |  |  |
|  | Refractometer |  |  |  |  |  |  |  | |  |  |
|  | Ophthalmoscope |  |  |  |  |  |  |  | |  |  |

Checklist for availability and utilization of medical equipments at **District Hospitals** (Radiology, lab, Dental, OR,, Medical, surgical, Gyn/Obs, ophthalmic dept.)

| S.No | Equipment name | Availability | | Number | Functioning status And Number | | | Utilization | | Monitoring |  |
| --- | --- | --- | --- | --- | --- | --- | --- | --- | --- | --- | --- |
|  |  | Yes | No |  | Functional | Not Functional | Not in use |  |  | Frequency | Agency |
|  |  |  |  |  |  |  |  | Yes | No |  |  |
|  | X-Ray |  |  |  |  |  |  |  |  |  |  |
|  | Arterial Blood Gas analyzer |  |  |  |  |  |  |  | |  |  |
|  | Electrosurgical Unit |  |  |  |  |  |  |  | |  |  |
|  | Autoclave |  |  |  |  |  |  |  | |  |  |
|  | Ultrasonic Washer |  |  |  |  |  |  |  | |  |  |
|  | Dental X-Ray Machine |  |  |  |  |  |  |  | |  |  |
|  | Ultrasound Machine |  |  |  |  |  |  |  | |  |  |
|  | Echocardiography Machine |  |  |  |  |  |  |  | |  |  |
|  | Patient Monitor |  |  |  |  |  |  |  | |  |  |
|  | Cardiac Monitor |  |  |  |  |  |  |  | |  |  |
|  | ECG Machine |  |  |  |  |  |  |  | |  |  |
|  | Defibrillator |  |  |  |  |  |  |  | |  |  |
|  | Anesthesia Machine |  |  |  |  |  |  |  | |  |  |
|  | Ventilator |  |  |  |  |  |  |  | |  |  |
|  | OT Table |  |  |  |  |  |  |  | |  |  |
|  | OT Light |  |  |  |  |  |  |  | |  |  |
|  | Suction Machine |  |  |  |  |  |  |  | |  |  |
|  | Syringe & Infusion Pump |  |  |  |  |  |  |  | |  |  |
|  | Infant Warmer |  |  |  |  |  |  |  | |  |  |
|  | Phototherapy Unit |  |  |  |  |  |  |  | |  |  |
|  | Fetal Doppler |  |  |  |  |  |  |  | |  |  |
|  | Pulse Oximeter |  |  |  |  |  |  |  | |  |  |
|  | ACT Machine |  |  |  |  |  |  |  | |  |  |
|  | Blood and Fluid Warmer |  |  |  |  |  |  |  | |  |  |
|  | Electromyogram Machine |  |  |  |  |  |  |  | |  |  |
|  | Electroencephalogram Machine |  |  |  |  |  |  |  | |  |  |
|  | Humidifier |  |  |  |  |  |  |  | |  |  |
|  | Pace Maker |  |  |  |  |  |  |  | |  |  |
|  | Infant Resuscitator |  |  |  |  |  |  |  | |  |  |
|  | Hot Pack Unit |  |  |  |  |  |  |  | |  |  |
|  | Traction Unit |  |  |  |  |  |  |  | |  |  |
|  | Continuous Passive Motion System |  |  |  |  |  |  |  | |  |  |
|  | Cold Pack unit |  |  |  |  |  |  |  | |  |  |
|  | Ultrasonic Tens System |  |  |  |  |  |  |  | |  |  |
|  | Microscopes |  |  |  |  |  |  |  | |  |  |
|  | Centrifuge/Cryofuge |  |  |  |  |  |  |  | |  |  |
|  | Hot Plate |  |  |  |  |  |  |  | |  |  |
|  | Cell Counter |  |  |  |  |  |  |  | |  |  |
|  | Cell Separator |  |  |  |  |  |  |  | |  |  |
|  | PH Meter |  |  |  |  |  |  |  | |  |  |
|  | Refrigerator |  |  |  |  |  |  |  | |  |  |
|  | Deep Freezer |  |  |  |  |  |  |  | |  |  |
|  | Bio-safety Cabinet |  |  |  |  |  |  |  | |  |  |
|  | Water Bath |  |  |  |  |  |  |  | |  |  |
|  | Laminar Flow |  |  |  |  |  |  |  | |  |  |
|  | Incubator |  |  |  |  |  |  |  | |  |  |
|  | Urine Analyzer |  |  |  |  |  |  |  | |  |  |
|  | Micropipettes |  |  |  |  |  |  |  | |  |  |
|  | Weighing Balance |  |  |  |  |  |  |  | |  |  |
|  | Plasma Thawing Bath |  |  |  |  |  |  |  | |  |  |
|  | Platelet Agitator |  |  |  |  |  |  |  | |  |  |
|  | Tube Sealer |  |  |  |  |  |  |  | |  |  |
|  | ELISA Reader |  |  |  |  |  |  |  | |  |  |
|  | Immuno Assay System |  |  |  |  |  |  |  | |  |  |
|  | Microtome |  |  |  |  |  |  |  | |  |  |
|  | Refractometer |  |  |  |  |  |  |  | |  |  |
|  | Ophthalmoscope |  |  |  |  |  |  |  | |  |  |
|  | Slit Lamp |  |  |  |  |  |  |  | |  |  |
|  | Keratometer |  |  |  |  |  |  |  | |  |  |
|  | Auto Perimeter |  |  |  |  |  |  |  | |  |  |
|  | Image Capturing system |  |  |  |  |  |  |  | |  |  |
|  | Dental Chair |  |  |  |  |  |  |  | |  |  |
|  | Dental Sterilizer |  |  |  |  |  |  |  | |  |  |
|  | Uroflowmeter |  |  |  |  |  |  |  | |  |  |
|  | ENT Examination Unit |  |  |  |  |  |  |  | |  |  |
|  | Harmonic Scalpel System |  |  |  |  |  |  |  | |  |  |
|  | Chest Vibrator |  |  |  |  |  |  |  | |  |  |
|  | Fibrillator |  |  |  |  |  |  |  | |  |  |
|  | VDRL Rotator |  |  |  |  |  |  |  | |  |  |
|  | Hormone Analyzer |  |  |  |  |  |  |  | |  |  |
|  | Air Sampler |  |  |  |  |  |  |  | |  |  |
|  | Wax Bath |  |  |  |  |  |  |  | |  |  |
|  | Surgical/Operating Microscope |  |  |  |  |  |  |  | |  |  |
|  | Phaco-emulsification Machine |  |  |  |  |  |  |  | |  |  |
|  | Tissue Flotation Bath |  |  |  |  |  |  |  | |  |  |
|  | Vortex Mixer |  |  |  |  |  |  |  | |  |  |
|  | Transport Incubator |  |  |  |  |  |  |  | |  |  |
|  | Refractometer |  |  |  |  |  |  |  | |  |  |
|  | Ophthalmoscope |  |  |  |  |  |  |  | |  |  |

**In-depth interview guide**

Name of the hospital ________________

Age of the interviewee_________

Sex ______________

Educational status _______

Role in the hospital ________________

1. What are the major sources of medical equipments of the hospital?

Probe – how are they obtained?

1. Are the products and technologies utilized and functioning appropriately?

Probe- per day/per patient or how

Probe- significance if equipment failure (frequency)

Probe- reason for high utilization (E.g. motivating factors, maintenance issue)

Probe – reason for less/no utilization

1. Does the hospital have sufficient human power to utilize/functionalize the existing medical devices?

Probe – to maintain and to monitor

Probe- timing for ordering equipment

Probe – any type of motivation

1. The involvements of appropriate professionals in the order, purchase, procure and utilize the products and technologies.
2. What quality assurance methods of medical device done?

Probe- frequency

Probe – by whom

Probe – how recommendations utilized.

Probe- presence of national guidelines, policies for ordering and procuring, maintaining

1. Solutions taken for non-functioning equipments
